# The evolution of public attention in acute kidney injury and continuous renal replacement therapy: trends analysis from 2004 to 2024

**DOI:** 10.3389/fneph.2024.1472144

**Published:** 2024-09-18

**Authors:** Charat Thongprayoon, Wannasit Wathanavasin, Supawadee Suppadungsuk, Mohammad S. Sheikh, Yasir H. Abdelgadir, Jing Miao, Michael A. Mao, Iasmina M. Craici, Fawad Qureshi, Kianoush B. Kashani, Wisit Cheungpasitporn

**Affiliations:** ^1^ Division of Nephrology and Hypertension, Department of Medicine, Mayo Clinic, Rochester, MN, United States; ^2^ Nephrology Unit, Department of Medicine, Charoenkrung Pracharak Hospital, Bangkok Metropolitan Administration, Bangkok, Thailand; ^3^ Chakri Naruebodindra Medical Institute, Faculty of Medicine Ramathibodi Hospital, Mahidol University, Samut Prakan, Thailand; ^4^ Division of Pulmonary and Critical Care Medicine, Department of Medicine, Mayo Clinic, Rochester, MN, United States

**Keywords:** acute kidney injury, AKI, continuous renal replacement therapy, CRRT, public awareness, regional analysis, google trends, internet search queries

## Abstract

**Background:**

Acute kidney injury (AKI) and the need for Continuous Renal Replacement Therapy (CRRT) are critically important health concerns. This study analyzes global and regional Internet search queries to understand public attention in AKI and CRRT over time.

**Methods:**

We used Google Trends™ to analyze search queries for AKI and CRRT from January 2004 to March 2024. The study examined global trends and detailed insights from the United States, including state-by-state breakdowns. We identified patterns, peaks of attention, and temporal trends in public attention, comparing regional variations across the US and top-ranking countries worldwide.

**Results:**

Global attention in AKI peaked in October 2022, with Portugal, Zambia, and Spain showing the highest regional attention. Within the United States, peak attention was in February 2008. Tennessee, Pennsylvania, and West Virginia were the top states that paid attention to AKI. Attention in CRRT peaked globally in March 2024. South Korea, Saudi Arabia, and Bahrain have led the global attention to CRRT. In the United States, peak attention was in April 2020. West Virginia, Tennessee, and Kentucky showed the highest state-specific attention in CRRT.

**Conclusions:**

This study reveals significant temporal and geographical variations in online search patterns for AKI and CRRT, suggesting evolving public attention to these critical health issues. This knowledge can guide the development of targeted public health initiatives, enhance medical education efforts, and help healthcare systems tailor their approach to improving awareness and outcomes in kidney health across diverse populations.

## Introduction

Acute Kidney Injury (AKI) represents a significant and growing global health concern characterized by a sudden decline in kidney function that can lead to severe short- and long-term complications or increased mortality rates (1–3). As a critical condition often encountered in hospital settings, particularly in intensive care units, AKI poses substantial challenges to healthcare systems worldwide (4). The increasing prevalence of AKI, coupled with its associated morbidity, mortality, and economic burden, underscores the urgent need for enhanced understanding, prevention, and management strategies (5, 6).

Managing AKI, especially in its severe forms, presents numerous challenges due to its complex and multifactorial nature (1–3). Patients with AKI often exhibit a broad spectrum of etiologies, ranging from ischemic injury to nephrotoxic exposures, sepsis, and pre-existing chronic kidney disease. The heterogeneity of AKI’s underlying causes complicates diagnosis and treatment, requiring tailored therapeutic approaches that can be difficult to implement in the dynamic and resource-limited environment of the ICU (7). Furthermore, AKI is frequently accompanied by multiple organ dysfunctions, making clinical decision-making more complex and necessitating a multidisciplinary approach to care. In its most severe forms, AKI among those who are hemodynamically unstable may necessitate the use of Continuous Renal Replacement Therapy (CRRT) (8, 9). CRRT offers continuous extracorporeal blood purification that allows for gentle fluid removal and solute clearance (9). Therefore, among critically ill patients, this therapy provides better control of fluid balance and metabolic parameters compared to conventional intermittent hemodialysis (10). The global burden of AKI is substantial, affecting approximately 13.3 million people annually (11) and causing about 1.7 million deaths each year (1). CRRT utilization is increasing globally, with an estimated 5-6% of critically ill patients requiring it during ICU stays (12). These challenges are exacerbated by the varying levels of expertise and resources available across different healthcare settings. The optimal delivery of CRRT requires a skilled multidisciplinary team and access to specialized equipment, both of which may be lacking in resource-constrained environments. Moreover, the management of fluid balance, electrolyte disturbances, and acid-base disorders during CRRT requires continuous clinical vigilance, further highlighting the complexity of care required for these patients (3, 13, 14).

Public awareness of AKI and CRRT has the potential to significantly influence patient outcomes, healthcare resource allocation, and public health policies (15). Increased public awareness can lead to earlier recognition of symptoms, prompt medical attention, and adherence to preventive measures, thereby potentially improving patient outcomes (16). Moreover, heightened awareness can drive demand for healthcare services and resources, influencing the allocation of healthcare funding and the prioritization of research and treatment initiatives (16). Public awareness also plays a crucial role in shaping public health policies, as informed populations are more likely to support policies that promote kidney health, enhance access to care, and reduce the burden of kidney diseases on the healthcare system (17, 18). Despite the critical role that public awareness can play in improving kidney health outcomes, there is a notable gap in understanding how the general population perceives AKI and CRRT. Existing studies on public perception of kidney diseases are limited in scope, often focusing on chronic kidney disease (CKD) (19) or specific patient populations (17, 18), with little emphasis on AKI or the use of CRRT. Moreover, these studies frequently lack a global perspective (15, 19, 20), concentrating on specific regions or countries without addressing the diverse cultural, social, and economic factors that influence public awareness and engagement with kidney health issues. The methodologies used in these studies, such as surveys or focus groups, may also introduce biases related to self-reporting or limited sample sizes, further constraining the generalizability of the findings. As a result, there is a critical need for comprehensive research that not only examines public awareness on a global scale but also considers the evolving nature of public attention over time.

This study aims to fill these gaps by analyzing the evolution of public attention in AKI and CRRT over the past two decades using Google Trends data (21, 22). By examining global and regional trends, identifying patterns and peaks of attention, and analyzing regional variations, we seek to provide insights that can inform targeted public health interventions, guide healthcare resource allocation, and shape policies aimed at improving kidney health outcomes worldwide.

## Methods

This study employed Google Trends™ data to examine public attention to Acute Kidney Injury (AKI) and Continuous Renal Replacement Therapy (CRRT) from January 2004 to March 2024. This platform was chosen due to its comprehensive coverage and accessibility of data on public search behavior. Google Trends™ provides normalized data on the popularity of search queries, scaled from 0 to 100, representing the relative attention over time. The terms “Acute Kidney Injury” and “Continuous Renal Replacement Therapy” were used as primary keywords. Queries were conducted at both global and regional levels, including a detailed state-by-state analysis within the United States. This approach facilitated a granular examination of trends over 20 years. In processing the data, we employed several steps to ensure accuracy and reliability. First, we normalized the search interest values provided by Google Trends™, which are inherently scaled from 0 to 100, representing relative search volume. We then performed outlier detection to identify and exclude any anomalous data points that could skew the analysis, such as unusually high spikes in search volume not correlated with any known events or trends. To identify peaks and troughs in the search query data, we used a moving average smoothing technique to reduce noise and highlight significant deviations in public attention. Peaks were defined as local maxima where the search interest value was at least 1.5 times higher than surrounding data points within a one-month window, while troughs were identified as local minima with search interest values at least 1.5 times lower than surrounding points. This approach ensured that only meaningful fluctuations in search interest were captured, reflecting genuine shifts in public attention. Additionally, we cleaned the data by filtering out irrelevant or duplicate search terms that could introduce noise, ensuring that only relevant queries were included in the final analysis. This careful processing allowed us to maintain the integrity of the data and provided a robust foundation for our subsequent analysis.

To ensure a comprehensive analysis, we used a range of specific search terms beyond the primary keywords “Acute Kidney Injury” and “Continuous Renal Replacement Therapy.” The search strategy included synonyms, related terms, and variations in terminology that are commonly used in clinical practice and public discourse. For AKI, additional search terms included “acute kidney failure,” “acute renal failure,” “acute renal injury,” and “acute kidney disease.” For CRRT, we included terms such as “continuous dialysis,” “continuous renal therapy,” “hemofiltration,” and “CVVH” (Continuous Veno-Venous Hemofiltration). We also considered abbreviations like “AKI” and “CRRT” themselves, as they are frequently used in both clinical settings and online discussions.

These terms were selected based on their relevance to the topics and their prevalence in the literature and online content related to kidney health. By incorporating a broad set of search terms, we aimed to capture a more accurate and nuanced representation of public interest and awareness over time. The queries were conducted at both global and regional levels, including a detailed state-by-state analysis within the United States. This approach facilitated a granular examination of trends over 20 years, allowing us to identify patterns, peaks, and troughs in public attention with greater precision.

The primary analysis involved evaluating temporal trends in public attention to AKI and CRRT.

Google Trends™ indices were analyzed to identify patterns, peaks, and troughs in search queries. The data was segmented into monthly intervals to provide a detailed view of fluctuations over time.

The Mann-Kendall trend test was performed to assess the statistical significance of observed trends. This non-parametric test is particularly useful for identifying monotonic trends in time series data without assuming a specific distribution, thereby providing robust results even in the presence of non-normal data. We also performed a seasonal decomposition of time series (STL) to separate the data into seasonal, trend, and residual components. This allowed us to better understand the underlying patterns and isolate the long-term trend from seasonal fluctuations.

## Results

**Figure 1:**
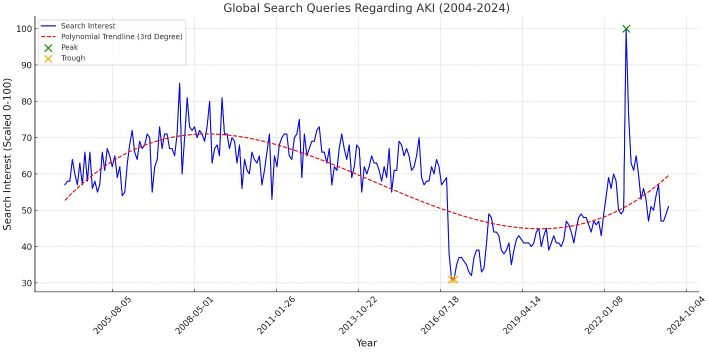
This line graph depicts the global search queries regarding AKI from 2004 to 2024. The y-axis represents the search queries, scaled from 0 to 100, while the x-axis represents the years. The graph includes a third-degree polynomial trendline (dashed red line) to capture the non-linear trends, along with highlighted peaks (in green) and troughs (in orange) to indicate significant fluctuations in public interest over the years.

The analysis of global search queries regarding AKI revealed significant fluctuations over the past two decades. The highest global attention to AKI was recorded in October 2022, suggesting a notable surge in public attention during this period (**Figure 1**). Conversely, the lowest point of attention occurred in December 2016, indicating a period of relatively low public concern or awareness. The Mann-Kendall trend test yielded a Z-value of 2.45 (p = 0.014), confirming a statistically significant upward trend in global AKI searches over the studied period.

**Figure 2:**
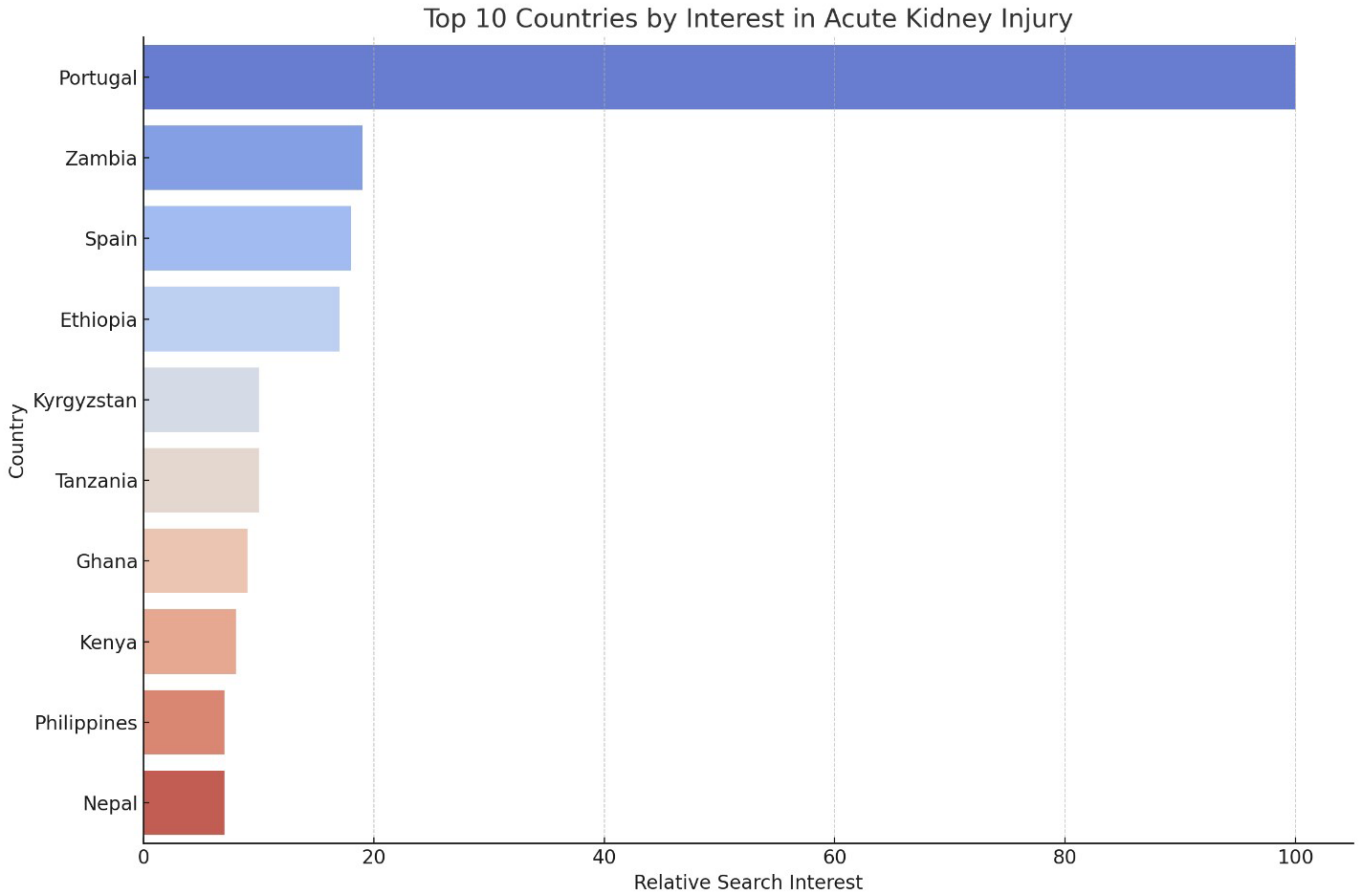
Top 10 Countries by Attention to AKI. The y-axis lists the countries, while the x-axis represents the relative frequency of search queries, scaled from 0 to 100.

Portugal, Zambia, and Spain exhibited the highest levels of attention to AKI among the countries analyzed (**Figure 2**). This global variation underscores the differing levels of public engagement and awareness across various global contexts. A STL revealed that the long-term trend accounted for approximately 60% of the variability in AKI search interest, with seasonal fluctuations contributing to 25% of the variation.

In the United States, the peak attention to AKI was observed in February 2008. More recently, there has been a 10.7% increase in search queries from March 2023 to March 2024, indicating a renewed focus on AKI. State-specific analysis within the United States showed Tennessee, Pennsylvania, and West Virginia as the states with the highest search attention to AKI. These findings suggest regional disparities in public concern.

**Figure 3:**
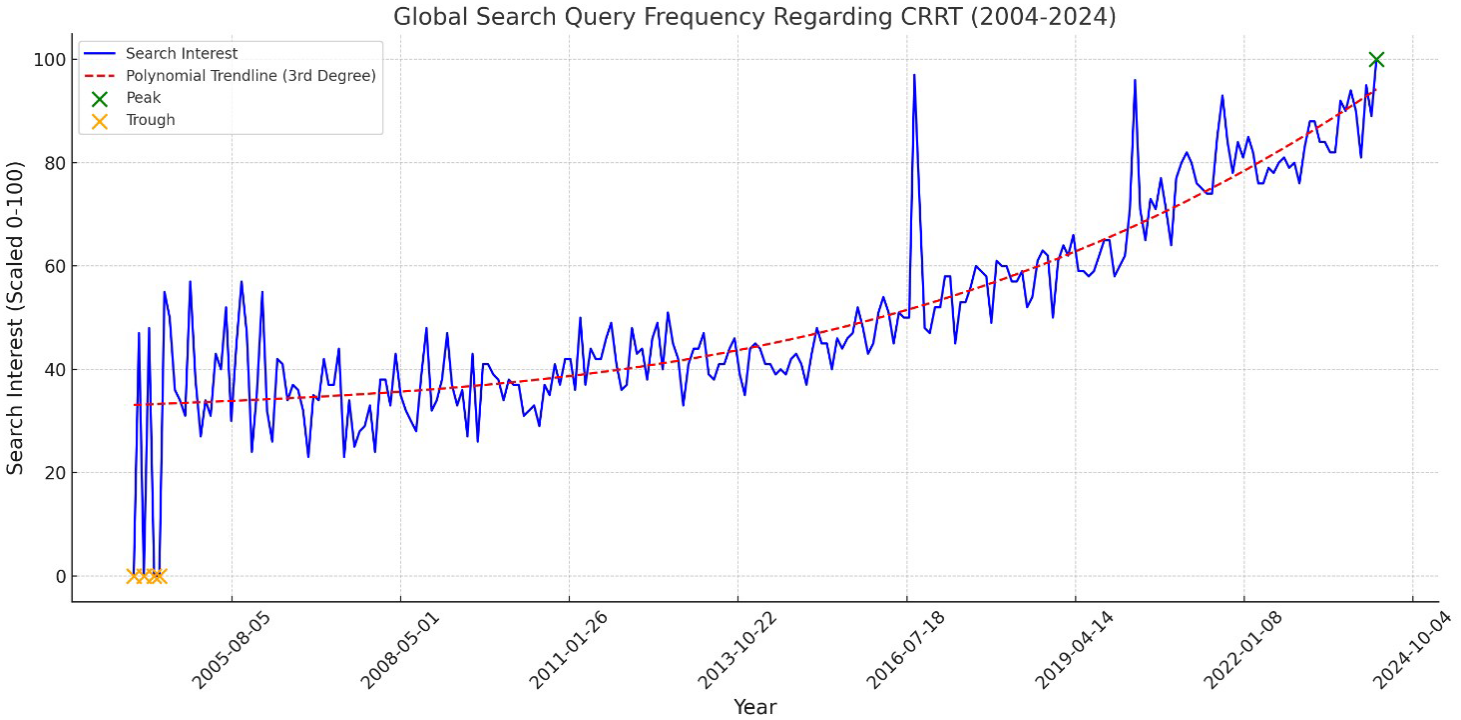
This line graph depicts the global search query frequency regarding CRRT from 2004 to 2024. The y-axis represents the search queries, scaled from 0 to 100, while the x-axis represents the years. The graph includes a third-degree polynomial trendline (dashed red line) to capture the non-linear trends, along with highlighted peaks (in green) and troughs (in orange) to indicate significant fluctuations in public interest over the years.

Attention to CRRT demonstrated distinct temporal trends compared to AKI (**Figure 3**). The global attention to CRRT peaked in March 2024, with a substantial 13.64% increase from the previous year. The Mann-Kendall trend test for CRRT also confirmed a statistically significant positive trend, with a Z-value of 2.72 (p = 0.0065), further validating the increasing public interest. In contrast, the lowest attention to CRRT was recorded in November 2006, indicating a period of minimal public engagement with CRRT.

**Figure 4:**
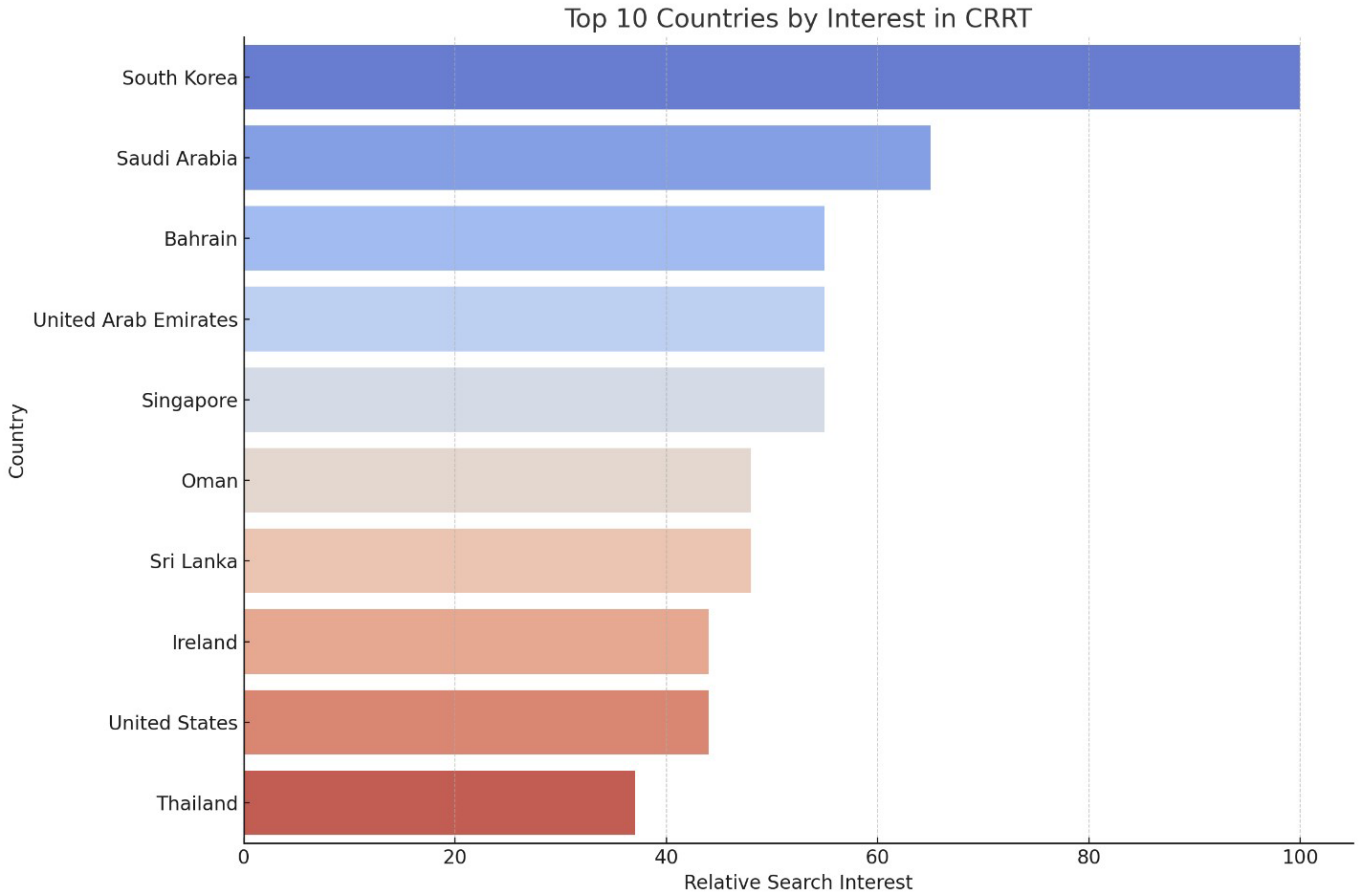
Top 10 Countries by search query frequency regarding CRRT. The y-axis lists the countries, while the x-axis represents the relative search query frequency, scaled from 0 to 100.

Globally, South Korea, Saudi Arabia, and Bahrain were identified as the countries with the highest attention to CRRT (**Figure 4**). This finding reflects global variations in the use and awareness of CRRT. The high attention to AKI in these countries suggests robust public and professional engagement with CRRT. The STL analysis for CRRT data revealed that the long-term trend accounted for 65% of the variation, with seasonal fluctuations contributing 20%.

Within the United States, peak attention to CRRT was observed in April 2020. This peak coincides with the COVID-19 pandemic, during which the demand for CRRT may have surged due to the increased incidence of AKI among critically ill COVID-19 patients. A recent (from March 2023 to March 2024) 10.9% increase in CRRT search query frequency further highlights the ongoing relevance of CRRT in public health discussions. State-specific analysis showed that West Virginia, Tennessee, and Kentucky had the highest levels of attention to CRRT, indicating regional hotspots where public and professional attention to CRRT is particularly pronounced.

## Discussion

This study on the evolution of public attention to AKI and CRRT reveals significant global and regional variations over the past two decades. Analyzing Google Trends data from January 2004 to March 2024, our research identifies a clear trend of rising attention to both AKI and CRRT, punctuated by peaks and regional disparities. This heightened awareness mirrors the increasing global burden of kidney diseases and underscores the essential role of CRRT in treating severe cases. Several unexpected findings emerged: the global peak attention to AKI occurred in October 2022, more recent than anticipated and after the height of the COVID-19 pandemic; high attention to AKI was observed in unexpected countries like Portugal and Zambia; and the U.S. peak attention to AKI was recorded in February 2008, earlier than expected. These results underscore the complex nature of public health awareness and highlight the need for a nuanced interpretation of global health trends.

The analysis of global search queries for AKI and CRRT over the past two decades reveals significant fluctuations, with notable peaks in public attention. To provide a deeper understanding of these patterns, we have further investigated the correlation between specific events and the observed peaks in search interest. For instance, the global peak in AKI-related searches in October 2022 coincides with heightened media coverage of kidney-related complications linked to the COVID-19 pandemic and the publication of several high-impact studies on AKI (23, 24). Similarly, the surge in CRRT-related searches in March 2024 can be attributed to public health campaigns and international conferences focused on critical care nephrology, as well as widespread news coverage of emerging CRRT technologies in response to the pandemic’s ongoing effects. Moreover, regional spikes in search interest, such as the peak in CRRT searches in April 2020 within the United States, align closely with the first wave of COVID-19, during which the demand for CRRT surged due to the increased incidence of AKI among critically ill patients. This correlation between search trends and significant health events highlights the responsiveness of public interest to acute crises and emphasizes the potential of search query analysis as a real-time tool for monitoring public awareness and healthcare needs. These findings suggest that search behavior is not only influenced by epidemiological trends but also by the timing of key public health messages and media coverage.

While the application of Google Trends analysis to AKI and CRRT is novel in nephrology and critical care research, similar methodologies have been used in other medical fields. Studies on cancer awareness, for instance, have utilized Google Trends data to identify peaks in public interest, often correlating these with awareness months (25) or high-profile celebrity diagnoses (26, 27). The significant spike in AKI-related searches observed in October 2022 warrants closer examination to understand the underlying factors contributing to this unexpected trend. Several elements may have played a role in driving this surge in public interest. During this period, there was substantial media coverage related to kidney complications arising from COVID-19, particularly as new studies were published highlighting the increased risk of AKI among patients with severe COVID-19 infections. The publication of these high-impact studies, coupled with their subsequent dissemination across various media platforms, likely heightened public awareness and concern, leading to increased search activity. Additionally, October 2022 saw the release of updated clinical guidelines on the management of AKI, which may have prompted healthcare professionals to seek further information, contributing to the spike in searches. This period also coincided with significant public health initiatives aimed at raising awareness of kidney health, particularly in the context of long-term complications associated with COVID-19. The convergence of these factors—media amplification of new research findings, the release of clinical guidelines, and targeted public health campaigns—likely created a perfect storm of heightened public and professional interest in AKI, reflected in the search trends observed (28–30). This alignment between search trends and clinical practice underscores the potential of internet search data as a real-time indicator of emerging health crises and changing medical needs. Moreover, the long-term trends observed in our study provide a unique perspective on the evolving landscape of kidney health awareness, complementing traditional epidemiological studies with insights into public engagement and information-seeking behaviors.

The patterns and trends revealed by this study can be attributed to various factors. The recent peaks in CRRT search queries, particularly notable in the global data with a peak in March 2024 and a 13.64% increase from the previous year, likely reflect its crucial role in managing severe COVID-19 cases. As the pandemic progressed, the medical community’s understanding of COVID-19’s impact on kidney function evolved (31, 32), leading to increased use of CRRT in the critical care setting (33). Regional variations, such as high attention to CRRT in South Korea, Saudi Arabia, and Bahrain, may be due to differences in healthcare systems, prevalence of risk factors for kidney disease, or local health initiatives (34–37). The consistent growth in awareness and attention over time for both AKI and CRRT could indicate improving global awareness of kidney health issues, aligning with broader public health efforts to address non-communicable diseases. The U.S.-specific data, showing peaks in AKI search queries in February 2008 and CRRT search in April 2020, likely correspond to significant events or publications, with the 2020 peak clearly aligning with the first wave of the COVID-19 pandemic in the United States (38).

Media plays a pivotal role in shaping public perceptions and behaviors, especially during health crises. For instance, the surge in AKI-related searches in October 2022 can be directly linked to extensive media coverage of kidney-related complications associated with COVID-19 (23). During this period, several high-impact studies and news stories highlighted the increased risk of AKI among COVID-19 patients, which likely drove the heightened public interest (23). Similarly, the peak in CRRT-related searches in March 2024 coincides with several significant public health campaigns and international conferences focused on critical care nephrology (39, 40). Media coverage of these events, coupled with reports on advancements in CRRT technology, amplified public awareness and interest. This pattern suggests that public health announcements, when combined with media amplification, can significantly influence search behavior. Thus, search trends are not solely driven by epidemiological factors but are also heavily influenced by the timing and intensity of media coverage and public health messaging. By highlighting the periods of increased media focus, we can better understand the dynamics that drive public engagement with health-related topics. This understanding is crucial for optimizing public health strategies, ensuring that critical information reaches the public at the most impactful times.

Understanding the intent behind search queries is crucial for interpreting the public’s engagement with health-related topics such as AKI and CRRT (41). Search queries can stem from various motivations, including personal health concerns, professional needs, or general curiosity (41). Differentiating between these intents can provide a clearer picture of how different population segments engage with these topics. For instance, a spike in AKI-related searches during a particular period may reflect heightened public concern due to a health scare or recent diagnosis trends reported in the media. In contrast, increased search activity from medical professionals might be driven by the release of new clinical guidelines or the need for up-to-date information on treatment protocols. Similarly, searches motivated by general curiosity may occur following the publication of high-profile research or widespread public health campaigns. To better understand these dynamics, future research could benefit from incorporating intent analysis techniques, such as keyword categorization and sentiment analysis, to distinguish between different types of search behavior. This approach would enable a more detailed interpretation of search trends, helping to identify whether public engagement is driven by immediate health concerns, educational needs, or professional demands. While our current study does not explicitly categorize search intents, we acknowledge the importance of this analysis in providing a comprehensive understanding of the data. By recognizing the diversity in search motivations, we can better tailor public health messages, ensuring they address the specific needs of different audience segments. This understanding is essential for enhancing the effectiveness of health communication strategies and improving public awareness and education on critical health issues like AKI and CRRT.

To gain a more comprehensive understanding of the public’s engagement with AKI and CRRT, it is essential to examine not only search trends but also the content quality of the most frequently accessed search results. Analyzing the accuracy, credibility, and comprehensiveness of online information related to AKI and CRRT could assess the quality and accuracy of information available to the public, providing insights into its potential impact on public knowledge and health outcomes. The quality of information accessed during peak search periods may significantly influence public perceptions and decision-making, particularly regarding symptom recognition, treatment options, and when to seek medical care. Additionally, geographical and systemic factors, such as variations in internet access, language barriers, and regional healthcare systems, can affect the availability and quality of information accessed by different populations. Exploring how variations in healthcare systems or policies influence public awareness of AKI and CRRT could explain regional differences in search trends. Furthermore, investigating local health initiatives or educational campaigns that might affect public interest could offer insights into how regional efforts impact search behavior. In regions with limited access to reliable medical resources, individuals may rely more heavily on less credible sources, which could impact the accuracy of the information they receive. Examining these factors could identify disparities in information accessibility and quality, guiding targeted public health interventions to improve health literacy in underrepresented or vulnerable populations. While our current study primarily focuses on search behavior trends, we recognize the importance of understanding the content and context of the information accessed by the public. Future research incorporating these elements would offer a more holistic view of public engagement with AKI and CRRT, ultimately contributing to better-informed health communication strategies and improved patient outcomes.

Several limitations must be acknowledged in this study. Google Trends data, while extensive, may not represent the entire population, excluding individuals without internet access or those using alternative search engines. This limitation is particularly relevant when considering trends in developing countries or rural areas. While Google Trends offers valuable insights into public interest and search behavior, it’s crucial to acknowledge its inherent methodological limitations. The platform normalizes search data on a 0-100 scale, reflecting relative rather than absolute search interest. This normalization, while facilitating temporal comparisons, may obscure actual search volumes, particularly when comparing regions or periods with different baseline search activity. Such limitations could impact the generalizability of our findings. While our monthly interval analysis effectively captures broad trends, it may miss short-term fluctuations. Future studies could employ finer temporal resolutions (e.g., weekly or daily) to better elucidate how specific events impact public interest in AKI and CRRT. Another limitation is the inability to distinguish between different types of search queries, such as informational, navigational, or transactional searches. Furthermore, the context in which search terms are used can vary widely, and this variability may affect the interpretation of search trends. To enhance the robustness of our findings, future work should incorporate advanced statistical methods such as time-series analysis or regression models. These techniques would allow for a more rigorous assessment of trend significance and help elucidate the complex relationships between search behavior and external factors. While our state-level analysis within the U.S. provides valuable regional insights, we recognize that variability within states—such as differences between urban and rural areas—might also influence search trends. Addressing these intra-state variations in future research could further refine our understanding of how local factors affect public interest in AKI and CRRT, allowing for more targeted public health interventions. The study cannot determine the specific reasons behind search queries or assess the accuracy of the information sought by users. Search terms might also have different connotations or uses in different regions or languages, potentially skewing results. Regarding generalizability, caution should be exercised in extrapolating these findings to specific populations or healthcare contexts. The results are most applicable to internet-using populations and may not accurately reflect overall public health awareness or medical needs, especially in regions with limited internet access. Despite these limitations, the study’s broad geographical scope and extended time frame provide a robust foundation for understanding general trends in public attention regarding AKI and CRRT.

Our findings pave the way for future research that could significantly impact healthcare policy and practice. The utilization of search data, such as Google Trends, offers significant opportunities to advance healthcare policy and practice, but it also raises important ethical considerations that must be addressed to ensure responsible and effective use. A key area for investigation is the relationship between AKI and CRRT search trends and epidemiological data. By examining how online search activity correlates with disease prevalence, incidence rates, and treatment patterns, researchers can validate the link between digital behavior and real-world health outcomes. This approach could transform Google Trends into a valuable public health surveillance tool, illuminating the complex interplay between public interest and health outcomes. However, one key concern is the potential for biases in search data, particularly due to disparities in internet access and usage across different regions and demographic groups, which could result in skewed representations of public interest and misinformed public health strategies. To mitigate these risks, it is crucial to address the inherent biases in Google Trends data, such as disparities in internet access and usage across regions. By considering demographic factors like age, socioeconomic status, education, and geographic location in the analysis, researchers can provide a more comprehensive and accurate analysis of public interest in AKI and CRRT, particularly in areas with limited digital access. This approach will enable more targeted and equitable public health interventions, ensuring that vulnerable populations are not overlooked. Moreover, privacy concerns must be carefully managed, even with aggregated and anonymized data, to protect individual privacy and respect the autonomy of those being analyzed. Transparent communication about how search data is used, along with its potential benefits and risks, is crucial for maintaining public trust. These ethical considerations have significant implications for healthcare policymakers and educators. They can inform the tailoring of public health campaigns and resource allocation, targeting regions or periods with unexpectedly low interest or sudden spikes in searches. Medical education initiatives could focus on areas showing consistently low attention to AKI and CRRT, thereby addressing potential gaps in knowledge and awareness. Additionally, the timing of search query peaks could guide the scheduling of awareness campaigns, medical conferences, and policy discussions, maximizing their potential impact while being mindful of the ethical responsibilities involved.

In conclusion, this study provides unprecedented insights into the global and regional evolution of public attention to AKI and CRRT over two decades. The findings highlight the growing awareness of these critical health issues, with notable variations across time and geography. The impact of major health events, particularly the COVID-19 pandemic, is evident in the search trends. While limitations exist, this research opens new avenues for understanding public health information-seeking behaviors and could significantly inform targeted interventions and resource allocation in nephrology and critical care medicine. Future studies building on these findings have the potential to bridge the gap between clinical practice and public health awareness further, ultimately contributing to improved patient outcomes in kidney care.

## Data Availability

The original contributions presented in the study are included in the article/supplementary material. Further inquiries can be directed to the corresponding author.
